# *Calr* type 1/like mutation in myelofibrosis is the most prominent predictor of momelotinib drug survival and longevity without transplant

**DOI:** 10.1038/s41408-024-01028-4

**Published:** 2024-03-19

**Authors:** Ayalew Tefferi, Animesh Pardanani, Kebede H. Begna, Aref Al-Kali, William J. Hogan, Mark R. Litzow, Rhett P. Ketterling, Kaaren K. Reichard, Naseema Gangat

**Affiliations:** 1 Division of Hematology, Mayo Clinic, Rochester, MN USA; 2 Division of Hematopathology, Mayo Clinic, Rochester, MN USA

**Keywords:** Myeloproliferative disease, Myeloproliferative disease

**TO THE EDITOR:**

Allogeneic hematopoietic stem cell transplant (AHSCT) is at present the only treatment modality in myelofibrosis (MF) that holds the promise of cure or prolongation of life [1]. Non-transplant treatment options in MF are palliative in scope and include Janus kinase 2 (JAK2) inhibitors (JAKi) [2]; FDA-approved, in this regard, are ruxolitinib (November 16, 2011), fedratinib (August 16, 2019), pacritinib (February 18, 2022), and most recently momelotinib (September 15, 2023) [3]. All four FDA-approved JAKi have proven to be variably effective in reducing spleen size and alleviating symptoms. In addition, momelotinib has distinguished itself, from ruxolitinib and fedratinib, by displaying an erythropoietic effect that has been attributed to the drug’s inhibitory activity on activin A receptor type-1 (ACVR1) [3–5]. Mechanistically, targeting ACVR1 interferes with SMAD signaling and regulation of transcription of genes that contributes to hepcidin expression, ineffective erythropoiesis, and terminal erythroid differentiation [6, 7].

The activity of momelotinib on all three major complications of MF, including anemia, splenomegaly, and constitutional symptoms, was first described more than 13 years ago by Pardanani et al., in the first-in-human phase-1/2 study, serially published between 2013 and 2023 [3, 8–12]. In a more recent account of the particular clinical trial, among 72 JAKi-naive MF patients who were anemic (hemoglobin level below the sex-adjusted reference range) at the time of treatment initiation with momelotinib, the authors reported anemia response in 44% (48% in those with baseline hemoglobin <10 g/dL) of patients and clinically vetted spleen response in 45% [11]. The unexpectedly favorable effect of momelotinib on anemia was subsequently confirmed by phase-2 [13, 14] and phase-3 [15] studies. In addition, we have recently reported long-term survival data among 183 clinical trial patients with MF, receiving one of several JAKi between 2007 and 2013, including ruxolitinib (*N* = 50), momelotinib (*N* = 79), fedratinib (*N* = 23), or BMS-911543 JAKi (*N* = 31) [10]; median post-JAKi survival was 3.7 years with 5/10-year survival rates of 41%/16% and drug discontinuation rate of 97%. In the particular report [10], survival was similar between the JAKi treatment cohorts, but drug survival was significantly longer for momelotinib vs. ruxolitinib (3-year drug discontinuation rate of 68% vs. 88%) [10, 12]; furthermore, 22 patients received AHSCT after failing JAKi therapy and enjoyed 5/10-year survival rates of 91%/45% vs. 47%/19% in the absence of AHSCT. In the current study, we focused on the 79 momelotinib-treated patients included in the aforementioned study, in order to identify molecular predictors of drug survival (i.e., drug retention) and longevity without AHSCT (transplant-censored survival).

This study was conducted under an institutional review board-approved minimum risk protocol that allowed retrospective collection and analysis of data from Mayo Clinic patients who participated in the above-referenced phase-1/2 core study of momelotinib (NCT00935987). Study design, drug doses and schedule, and additional details on treatment effect and side effects have previously been published [9]. The current study is restricted to analysis of patients who were JAKi-naive prior to their treatment with momelotinib and is focused on drug survival (i.e., drug retention) and transplant-censored survival; the former was calculated from time of initiation of momelotinib to time of treatment discontinuation while the latter required censoring of patients at the time of AHSCT. Mutation information used in the current study, other than those involving MPN-specific driver mutations (i.e., *JAK2*, *CALR*, *MPL*), was restricted to those performed under a research protocol and did not include additional routine NGS-derived data, as was the case in some of our prior studies [10–12]. Anemia and spleen response criteria for the current study were according to the revised international working group for MPN research and treatment (IWG-MRT) criteria, with some modifications [16]; MRI/CT confirmation of clinically vetted spleen response was not required. Age cutoff levels used for survival analysis were determined by receiver operating characteristic (ROC) curve analysis. Conventional statistical methods were applied using JMP Pro 16.0.0 software (SAS Institute, Cary, NC, USA).

**Fig. 1:**
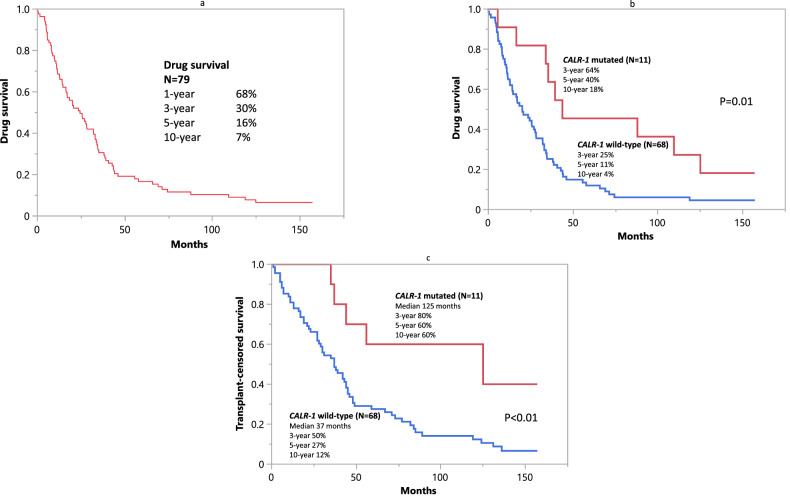
Momelotinib drug survival and transplant-censored survival data. **a** Drug survival among 79 JAK inhibitor-naive patients with myelofibrosis. **b** Drug survival among 79 JAK inhibitor-naive patients with myelofibrosis stratified by *CALR* type-1/like (*CALR*-1) mutation. **c** Transplant-censored survival among 79 JAK inhibitor-naive patients with myelofibrosis treated with momelotinib and stratified by *CALR* type 1/like (*CALR*-1) mutation.

A total of 79 JAKi-naive patients with MF were considered: median age 67 years; males 54%; MF variant PMF 65%, post-PV MF 20%, post-ET MF 15%; driver mutation distribution *JAK2* 71%, type-1/like *CALR* (*CALR*-1) 14%, type 2/like *CALR* (*CALR*-2) 3%, *MPL* 8%, triple-negative 5% (Table 1). Risk category according to the dynamic international prognostic scoring system (DIPSS) [17] was high/intermediate in 80% of the patients and unfavorable karyotype was documented in 29%. Information on other mutations performed under the research protocol was available in 45 cases and included *ASXL1* 22%, *SRSF2* 18%, and either *ASXL1* or *SRSF2* in 38% (Table 1). The median time from the initial diagnosis to the time of study entry was 25.2 months, and patients were enrolled between November 2009 and March 2011 with follow-up information updated through July 2023; during this period, 68 (86%) deaths, 13 (16%) leukemic transformations, and 7 (9%) AHSCTs were recorded; median follow-up for alive patients was 12.5 years (range 11.3–13.1; Table 1). As previously noted in our previously published report [11], the anemia response rate in patients with baseline hemoglobin level of <10 g/dL was 48%, and the spleen response rate 47%. The median duration of treatment with momelotinib was 25 months (range 0.1–157); the proportion of patients remaining on momelotinib treatment was 68% at 1 year, 30% at 3 years, 16% at 5 years, and 7% at 10 years (Fig. 1a). At the time of this writing, 5 (6.3%) of the original 79 patients were still receiving momelotinib therapy with treatment durations between 152+ to 157+ months; these 5 patients make up 45% of the 11 patients currently alive; among the remaining 6 alive patients, 5 had received AHSCT.

**Table 1:** Clinical and laboratory characteristics of 79 JAK inhibitor treatment-naive patients with myelofibrosis obtained at the time of momelotinib study entry, stratified by whether or not they remained on the study drug for at least 5 years.

Variables	All patients (*N* = 79)	Momelotinib for ≥ 5 years (*N* = 24)	Momelotinib for < 5 years (*N* = 55)	*P* value
Median age in years (range)	67 (34–89)	65 (34–74)	68 (48–89)	**0.01**
Age >65 years: *N* (%)		11 (46)	34 (62)	0.2
Males: *N* (%)	43 (54%)	11 (46)	32 (58)	0.3
MF variant: *N* (%)				
PMF	51 (65)	14 (58)	37 (67)	0.7
Post-PV MF	16 (20)	6 (25)	10 (18)	
Post-ET MF	12 (15)	4 (17)	8 (15)	
Type-1/like *CALR*	11 (14)	7 (29)	4 (7)	**0.01**
Driver mutations: *N* (%)				
* JAK2*	56 (71)	15 (63)	41 (75)	0.1
* CALR-1*	11 (14)	7 (29)	4 (7)	
* CALR-2*	2 (3%)	0	2 (4)	
* MPL*	6 (8)	1 (4)	5 (9)	
Triple-negative	4 (5)	1 (4)	3 (5)	
DIPSS high/intermediate-2 risk category: *N* (%)	64 (80)	16 (64)	48 (88)	**0.02**
Hemoglobin <10 g/dl: *N* (%)	54 (68)	16 (67)	38 (69)	0.8
Leukocyte count >25 ×10^9^/L: *N* (%)	19 (24)	3 (13)	16 (29)	0.1
Platelet count <100 × 10^9^/L: *N* (%)	19 (24)	6 (25)	13 (24)	0.9
Circulating blasts ≥1%: *N* (%)	58 (73)	16 (67)	42 (76)	0.4
Constitutional symptoms: *N* (%)	46 (58)			
Unfavorable karyotype: *N* (%)	23 (29)	3 (13)	20 (36)	**0.02**
*ASXL1* mutation—*N evaluable* = *45*	10 (22)	2 (18)	8 (24)	0.7
*SRSF2* mutation—*N evaluable* = *45*	8 (18)	2 (18)	6 (18)	0.9
*ASXL1* or *SRSF2* mutation—*N evaluable* = *45*	17 (38)	4 (36)	13 (38)	0.9
Median time diagnosis to study entry: months	25.2	15.3	26	0.74
Median f/u for living patients: years (range)	12.5 (11.3–13.1)	12.6 (11.3–13.1)	11.6 (11.45–11.7)	0.2
Deaths: *N* (%)	68 (86)	15 (63)	53 (96)	**<0.01**
Leukemic transformation: *N* (%)	13 (16)	3 (13)	10 (18)	0.5
Allogenic transplant: *N* (%)	7 (9)	4 (17)	3 (6)	0.12
Median time on treatment in months (range)	25 (0.1–157)	68 (38–157)	14 (0.1–35)	**<0.01**
Anemia response: *N* (%)—*N evaluable* = *54*	26 (48)	9 (56)	17 (45)	0.4
Spleen response: *N* (%)—*N evaluable* = *70*	33 (47)	16 (76)	17 (35)	**<0.01**

*MF* myelofibrosis, *PMF* primary MF, *PV* polycythemia vera, *ET* essential thrombocythemia, *DIPSS* dynamic international prognostic score.

Bold font highlights statistically significant values.

In univariate analysis, molecular predictors of drug survival included *CALR*-1 (*P* = 0.006) but not *ASXL1* (*P* = 0.6), *SRSF2* (*P* = 0.3) or any other myeloid neoplasm-related gene mutation evaluated; unfavorable karyotype was of borderline significance (*P* = 0.08). Of note, among the 11 *CALR*-1 mutated patients, 8 had PMF and 3 post-ET MF; the impact of *CALR*-1 mutation on overall and drug survival was not differentially affected in PMF vs. post-ET MF, with the caveat that the number of informative cases in the latter instance was too small to be definitive (data not shown). The 3-, 5-, and 10-year drug survival rates in *CALR*-1-mutated patients were 64%, 40%, and 18%, respectively, vs. 25%, 11%, and 4%, in *CALR*-1 wild-type (*P* = 0.01, Fig. 1b). In multivariable analysis, *CALR*-1 (HR 0.4, 95% CI 0.2–0.87), age <63 years (HR 0.5, 95% CI 0.3–0.89), and absence of baseline transfusion dependency (HR 0.6, 95% CI 0.3–0.9) were associated with superior drug survival. In addition, spleen response (*P* < 0.01), but not anemia response (*P* = 0.23), was associated with longer drug survival; in multivariable analysis *CALR*-1, age, and spleen response retained their significance. These observations were also apparent when comparing patients on treatment for ≥5 vs. <5 years (Table 1).

Multivariable analysis of genetic variables for transplant-censored survival identified absence of *CALR*-1 (HR 3.5, 95% CI 1.2–10.5; *P* = 0.03) and presence of high-molecular-risk (HMR; *ASXL1*/*SRSF2*) mutations (HR 2.3, 95% CI 1.1–4.6; *P* = 02), but not unfavorable karyotype (*P* = 0.3) to be predictive of transplant-censored inferior survival. Among 11 patients with *CALR*-1 mutation, median transplant-censored survival was 125 months with 10-year survival rate of 60% (Fig. 1c); by contrast, the corresponding figures were 37 months and 12%, in the 68 patients not harboring *CALR*-1 mutation (Fig. 1c). Combination of *CALR*-1 and HMR mutational states enabled further risk stratification but the numbers of informative cases in each genetic subgroup were too small to warrant graphic depictions. Regardless, in multivariable analysis, absence of *CALR*-1 (HR 2.9, 95% CI 1.02–8.1), age ≥63 years (HR 3.0, 95% CI 1.5–6.0), and absence of spleen response (HR 2.6, 95% CI 1.5–4.5) were identified as being most significant in predicting inferior transplant-censored survival.

Survival in MF is currently determined by the presence or absence of several genetic and clinical risk factors that are assembled into contemporary risk models, including the mutation- and karyotype-enhanced international prognostic system, version 2.0 (MIPSS*v2*) [18]. MIPSS*v2* risk factors include very high risk (VHR)/unfavorable karyotype, HMR mutations, absence of *CALR*-1 mutation, constitutional symptoms, anemia, and circulating blasts ≥2%; based on these factors, 10-year survival estimates range from 0 to 10% for very high/high to 50-86% for low/very low-risk disease [18]. Accordingly, it was not surprising that the current study found *CALR*-1 mutation to be the most important risk factor for both drug survival and longevity without AHSCT, in momelotinib-treated patients with MF. Such information might help select MF patients who might benefit from treatment with momelotinib and in whom AHSCT might be deferred. On the other hand, median survival in the absence of *CALR-1* mutation and presence of HMR mutation (*N* = 19) was only 19 months with no patient surviving beyond 7 years and thus warranting upfront consideration of AHSCT in such patients. In the absence of both *CALR-1* and HMR mutations, median survival was 33 months, and timing of AHSCT is best determined by the presence or absence of other risk factors, as outlined above [18]. The findings in the current study should not distract from the fact that the therapeutic value of momelotinib, as is the case with other JAKi, is limited to the palliation of symptoms and that our MF patients remain in dire need of disease-modifying drugs.
